# Thermally Conductive Biopolymers in Regenerative Medicine and Oncology: A Systematic Review

**DOI:** 10.3390/ph18111708

**Published:** 2025-11-11

**Authors:** Ivett Poma-Paredes, Oscar Vivanco-Galván, Darwin Castillo-Malla, Yuliana Jiménez-Gaona

**Affiliations:** 1 Departamento de Química y Ciencias Exactas, Universidad Técnica Particular de Loja, San Cayetano Alto s/n, Loja CP1101608, Ecuador; mipoma1@utpl.edu.ec (I.P.-P.); dpcastillo@utpl.edu.ec (D.C.-M.); 2 Departamento de Ciencias Biológicas y Agropecuarias, Universidad Técnica Particular de Loja, San Cayetano Alto s/n, Loja CP1101608, Ecuador; oavivanco@utpl.edu.ec

**Keywords:** hyperthermia, thermal therapy, biopolymers, regenerative medicine, biocompatibility

## Abstract

**Background:** Minimally invasive hyperthermia and regenerative therapies require materials that deliver precise, localized heat without compromising biocompatibility. Most conventional polymers are thermally insulating and challenging to control in vivo, motivating this review. **Objectives:** We aimed to (i) examine the use of thermally enhanced biopolymers in hyperthermia-based therapies, (ii) appraise evidence from clinical and preclinical studies, (iii) identify and classify principal applications in regenerative medicine. **Methods:** A PRISMA-guided systematic review (2020–2025) with predefined inclusion/exclusion criteria was conducted and complemented by a bibliometric analysis using VOSviewer for mapping and visualization. **Results:** Modifying biopolymers—via functionalization with photothermal or magnetic nanoagents (Au; Fe_2_O_3_/Fe_3_O_4_/CoFe_2_O_4_; CuS; Ag; MXenes, e.g., Nb_2_C), crosslinking strategies, and hybrid formulations—significantly increased thermal conductivity, enabling localized hyperthermia and controlled drug release. In vitro and in vivo studies showed that europium-doped iron oxide nanoparticles embedded in chitosan generated heat efficiently while sparing healthy tissues, underscoring the need to balance biocompatibility and thermal performance. Hydrogel systems enriched with carbon nanomaterials (graphene, carbon nanotubes) and matrices such as GelMA, PNIPAM, hyaluronic acid, and PLA/PLGA demonstrated tissue compatibility and effective thermal behavior; graphene was compatible with neural tissue without inducing inflammation. **Conclusions:** Thermally conductive biopolymers show growing potential for oncology and regenerative medicine. The evidence supports further academic and interdisciplinary research to optimize safety, performance, and translational pathways.

## 1. Introduction

Advancing biopolymer research is essential for designing sustainable biomaterials with applications in tissue engineering, drug delivery, and regenerative medicine. In this sense, oncology and regenerative medicine are receiving attention through the recent development of biopolymers with high thermal conductivity, which allow minimally invasive interventions with precise temperature control.

In this context, this review covers (i) what thermally conductive biopolymers are and how they are obtained; (ii) which physical and chemical properties govern their performance; (iii) how molecular structure influences heat transfer; (iv) which mechanisms of action drive their thermal response. We focus on advances from 2020 to 2025 to ensure a contemporary scope.

Recent reviews, such as those by Bala et al. [1] and Szwed and Marczak [2], examine the applications of biopolymers in oncology, with particular emphasis on hyperthermia and tumor ablation. In addition, they identify and categorize applications in regenerative medicine—such as tissue engineering and controlled release systems—underscoring their translational potential [1,2,3,4].

In terms of materials, biodegradable biopolymers of polysaccharide, protein, and aliphatic origin are considered alginate, chitosan, polylactic acid, and fibroin, whose linked monomers (glycosidic, peptide, or ester) carry functional groups with high affinities for water and remarkable chemical modification capacity. These materials can be doped with nanoparticles (e.g., Fe_3_O_4_, CoFe_2_O_4_, plasmonic Au, MXenes) to modify thermal conductivity, strengthen mechanical integrity, and enable bioactive and controlled release functions [5,6,7].

The mechanisms underlying their performance in hyperthermia include magnetic losses due to Néel and Brown relaxation in nanoparticles subjected to alternating fields, as well as efficiency quantification using parameters such as Specific Absorption Rate (SAR) and Initial Loss Parameter (ILP); these interactions promote apoptosis and selective necrosis, oxidative stress, and activation of heat shock proteins, with complementary immunostimulatory effects [1,2,8].

In oncology, preclinical evidence shows a significant reduction in tumor viability with systems based on Fe_3_O_4_ doped or coated with biopolymers and photothermal platforms directed by hyaluronic acid [9,10,11]. Clinically, modulated hyperthermia combined with chemotherapy improves tumor control in pancreatic cancer [4], and coated superparamagnetic formulations (SPIONs) have reached clinical phases in glioblastoma with a good safety profile and survival benefit, even materializing in a medical device (Szwed & Marczak [2]). These trajectories confirm the value of thermal strategies combined with radio- or chemotherapy.

In regenerative medicine, poly (lactic-co-glycolic acid) (PLGA), β-tricalcium phosphate (β-TCP), three-dimensional (3D) scaffolds incorporating MXenes (two-dimensional transition-metal carbides/nitrides—e.g., niobium carbide, Nb_2_C), and poly(ε-caprolactone) (PCL) structures decorated with plasmonic gold nanoparticles enabled mild, controlled hyperthermia (≈41–42 °C) that activated angiogenic and osteogenic pathways, accelerating vascularized bone formation. Oxidized hyaluronic acid (OHA)/polyaniline hydrogels display self-healing capability and sustained nitric oxide (NO) release, promoting antibacterial and regenerative responses. Likewise, silk fibroin– or tragacanth-based composites incorporating magnetite (Fe_3_O_4_) and alginate have been explored for improved mechanical and biological performance [12,13,14].

A critical reading of the field reveals recurring translation bottlenecks. Recent reviews on layered double hydroxides (LDHs) for regenerative nanomedicine explicitly note that clinical translation remains in its infancy, citing unmet needs in standardized synthesis/characterization, biosafety, long-term stability, drug-loading control, and clear pathways from bench to clinic, all of which complicate reproducibility and cross-study comparison when thermal functions are engineered into biopolymers [15].

Likewise, a review on hydrogel [16] scaffolds for dental-pulp regeneration highlights heterogeneity in mechanical/viscoelastic properties, immune interactions, and sterilization/processing, as well as a scarcity of well-designed clinical studies, underscoring that in vitro efficacy rarely maps cleanly onto complex tissue microenvironments where heat transfer and perfusion vary spatiotemporally.

Finally, the report in [17] illustrates another pervasive gap: bench-skewed evidence (e.g., physicochemical profiling and anti-tumor effects primarily in vitro) with limited in vivo pharmacokinetics/biodegradation and no harmonized thermometry or dosimetry (SAR/ILP) standards makes it challenging to generalize safety windows, predict off-target heating, or satisfy regulatory expectations for hyperthermia-enabled biomaterials.

The paper is organized as follows: Section 1 presents related and interdisciplinary works and real research challenges. Section 2 describes the workflow of the methodology. Section 3 describes the results of the review. Section 4 discusses the findings, and Section 5 provides conclusions, limitations, and implications for practice and future research.

### Related Work

The intersection of thermally conductive biopolymers with oncology and regenerative medicine has grown rapidly. A consistent theme is the dual role of these materials, enhancing hyperthermia-based cancer treatments and enabling functional scaffolds for tissue engineering. In oncology, several authors highlight the potential of biopolymer–nanoparticle composites in localized thermal therapies; e.g., Bala et al. [1] and Szwed and Marczak [2] underscore the significance of integrating biopolymers with magnetic or photothermal nanoagents to improve tumor ablation efficiency while maintaining biocompatibility.

Dragojevic et al. [3] and Fiorentini et al. [4] provide clinical perspectives, reporting that modulated hyperthermia combined with chemotherapy enhances tumor control in pancreatic and brain cancers. At the same time, superparamagnetic iron oxide nanoparticles (SPIONs) coated with biopolymers have advanced into clinical evaluation for glioblastoma, demonstrating both safety and survival benefits.

From a materials perspective, polysaccharide- and protein-based matrices such as alginate, chitosan, and fibroin are recurrently cited for their adaptability and capacity for chemical modification [5,6,7]. Their functionalization with nanoparticles such as Fe_3_O_4_, CoFe_2_O_4_, and Au enhances thermal conductivity and mechanical stability while enabling synergistic mechanisms, such as apoptosis induction, oxidative stress modulation, and activation of heat shock proteins [8]. These findings align with the paradigm that thermal strategies in oncology are most effective when combined with conventional therapies, thus reinforcing their translational value.

In regenerative medicine, research increasingly focuses on biopolymer scaffolds functionalized with conductive nanomaterials to promote tissue repair. Some studies report that PLGA/β-TCP scaffolds doped with MXenes (Nb_2_C) and PCL matrices embedded with plasmonic gold nanoparticles enable controlled hyperthermia (~41–42 °C), stimulating angiogenesis and osteogenesis [18,19]. Hydrogels enriched with conductive components, such as OHA/polyaniline and fibroin/tragacanth composites, which exhibit antibacterial activity, self-repair properties, and enhanced nutrient diffusion, ultimately supporting cell viability and wound healing [20,21]. Alginate-based systems illustrate therapeutic delivery and regenerative applications [22].

Despite the advances, key challenges remain before full clinical translation. As highlighted by Fiorentini et al. [4] and Szwed and Marczak [2], the transition from preclinical promise to clinical application requires continued investigation into long-term safety, optimized formulations, and scalable manufacturing.

Firstly, most studies are limited to in vitro or small-animal models, with relatively few large-scale preclinical or clinical trials [2,4]. This raises concerns about the reproducibility of hyperthermia outcomes in heterogeneous human tumors and across diverse tissue environments.

Second, while nanoparticle functionalization strategies (e.g., Au, Fe_3_O_4_, MXenes) have enhanced thermal conductivity and biocompatibility, long-term toxicity, clearance, and biodistribution issues remain underexplored [5,8]. Although effective, carbon-based nanomaterials such as graphene [23] and nanotubes still face questions regarding immune response modulation and chronic inflammation risk [9].

Third, most regenerative applications have focused on bone and wound healing, with limited exploration of neural, cardiac, or soft tissue regeneration, where precise thermal modulation could have substantial therapeutic benefits [18,19]. Moreover, integrating innovative feedback systems, biosensors capable of real-time monitoring of temperature, pH, or metabolic markers, remains conceptual, though it represents a key step toward clinically viable “intelligent” biomaterials.

Based on the previous research described above, the main aims of this research are as follows: (i) to examine the use of thermally enhanced biopolymers in hyperthermia-based therapies, (ii) to appraise evidence from clinical and preclinical studies, (iii) to identify and classify principal applications in regenerative medicine.

## 2. Materials and Methods

This review followed the PRISMA methodology as follows: (i) Using inclusion/exclusion criteria, data retrieval, and extraction. (ii) Identifying relevant studies from scientific databases (Scopus) using defining variables and Logical Booleans search connectors. (iii) Analyzing bibliometric mapping (co-occurrence networks: nodes, size, and connections) using selected scientific data with the VOSviewer tool (v1.6.20) van Eck et al. [24] (iv) Conducting quality assessment and obtaining graphical statistics of studies using AI-assisted bibliometric analysis for synthesis and thematic trends.

### 2.1. PRISMA Methodology

**Figure 1:**
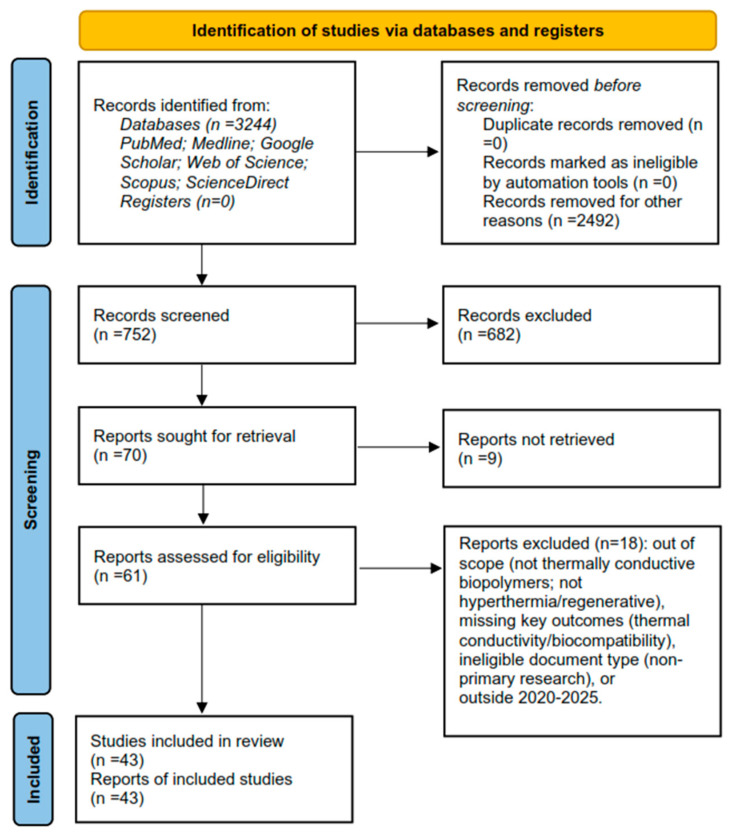
PRISMA 2020 flow diagram of the study-selection process (databases and registers).

According to the methodology proposed by Khan et al. [25] and the protocol proposed by Moher et al. [26], a checklist was established to guide the conduct of systematic reviews. The checklist covers fundamental aspects such as identification of relevant studies, eligibility criteria, search strategy variables, and bibliometric map, among others. The research process is shown in Figure 1; it followed the flowchart and the PRISMA protocol. This systematic review was registered in PROSPERO CRD420251165632 (https://www.crd.york.ac.uk/PROSPERO/view/CRD420251165632, accessed on 10 October 2025).

#### 2.1.1. Identification of Relevant Studies

An exhaustive search was conducted in academic databases such as Scopus, PubMed, Science Direct, Medline, Web of Science, and ScienceDirect (publisher platform for full texts). We additionally screened Google Scholar (first 200 results by relevance) and major conference proceedings in biomaterials/hyperthermia (e.g., SPIE/IEEE Xplore, as well as relevant conferences on biomaterials, hyperthermia, and thermally conductive biopolymers,) to identify studies not indexed elsewhere. Keywords were used, such as “thermally conductive biopolymers”, “hyperthermia in tumor treatment”, “regenerative medicine”, “biocompatibility of biopolymers”, and “thermal properties of biomaterials”.

The steps carried out while applying the PRISMA methodology are described below.

#### 2.1.2. Inclusion Criteria

(i) Original clinical or preclinical (in vitro/in vivo) studies;
(ii) Thermally conductive biopolymers used in hyperthermia and/or regenerative/cell-regeneration contexts;
(iii) Outcomes on thermal performance (e.g., conductivity, SAR/ILP, temperature rise) and biocompatibility/safety;
(iv) Publication years 2020–2025;
(v) Data extractable for qualitative synthesis.

#### 2.1.3. Exclusion Criteria

(i) Studies not centered on thermally conductive biopolymers;
(ii) Studies unrelated to hyperthermia or regenerative applications;
(iii) Studies lacking relevant thermal or biocompatibility data;
(iv) Non-original items (editorials, letters, narrative reviews, conference abstracts without complete data);
(v) Duplicates;
(vi) Records outside 2020–2025.

*Timeframe*. The search covered 13 April 2024 to 13 February 2025.

*Study selection and counts.* The Scopus query retrieved 3244 records; after year/keyword filters, this was reduced to 752. Following aggregation across all sources and deduplication, 43 studies met the inclusion criteria and were retained for synthesis (see PRISMA 2020 flow diagram, Figure 1).

*Data extraction.* We extracted data on material type and formulation (polymer class, crosslinking/functionalization, nanoagent), thermal parameters (conductivity, SAR/ILP with field parameters, CEM43 when available), biocompatibility/safety (cytotoxicity, histology, immune markers), model (in vitro/in vivo/clinical), outcomes, and study design features.

*Bibliometrics.* For VOSviewer mapping (co-occurrence), we used fractional counting with a minimum term occurrence threshold (≥5), unified synonyms, and reported cluster structure (nodes, links, total link strength) to visualize thematic trends.

#### 2.1.4. Selection of Bibliographic Search Variables

Information obtained from public and private databases about biopolymers, thermal properties, and biocompatibility was analyzed using bibliographic methods to understand the use of these materials in biomedical applications.

Table 1 shows the keywords and logical connectors (AND, OR) used to perform more specific searches of the terminology applied in this study. In addition, it provides guidance on the process of study selection, data extraction, and risk of bias assessment, as well as the evaluation of the certainty of the evidence. These keywords were applied to reviews focusing on clinical interventions, tissue engineering, and controlled drug delivery, enabling the inclusion of both quantitative and qualitative studies [27].

#### 2.1.5. Data Selection

The previously described keywords were used to generate a search in the Scopus database (https://www.scopus.com/, accessed on 10 June 2025). The search yielded 3244 indexed articles, and after applying filters by year and keywords, 752 articles were obtained. However, the most relevant studies (43 documents) were selected according to the inclusion and exclusion criteria previously described. Then, the files were filtered and exported in .csv format to upload to the VOSviewer (v1.6.20) software for creating and visualizing maps based on network data.

#### 2.1.6. Bibliometric Map

The search in Scopus using the terms “biopolymers” AND “medical” AND “applications” yielded 3244 initial records. After applying filters for document type (articles and reviews), period 2020–2025, and keywords, the set was reduced to 752 documents exported in CSV format for analysis. The files were processed in VOSviewer (v1.6.20) via the *Create a map based on bibliographic data* module, constructing a co-occurrence network with unit of analysis = author keywords, counting method = complete counting, and a minimum occurrence threshold = ≥5 per term. A thesaurus was applied to normalize lexical variants (singular/plural, hyphenation, and spelling). Under these parameters, *n* = 74 terms met the threshold and were included in the network (*Keywords in the map appear in English because they correspond to the author-assigned terms as indexed in Scopus*). The available options, selected parameters, and the settings applied in this study are summarized in Table 2.

##### Bibliometric Analysis

**Figure 2:**
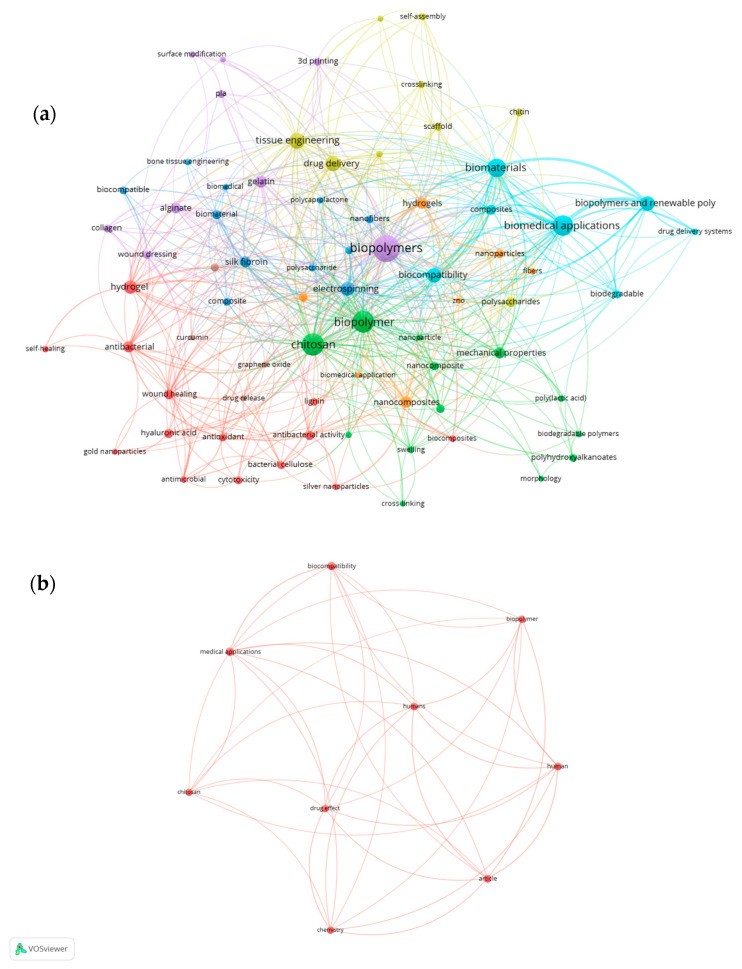

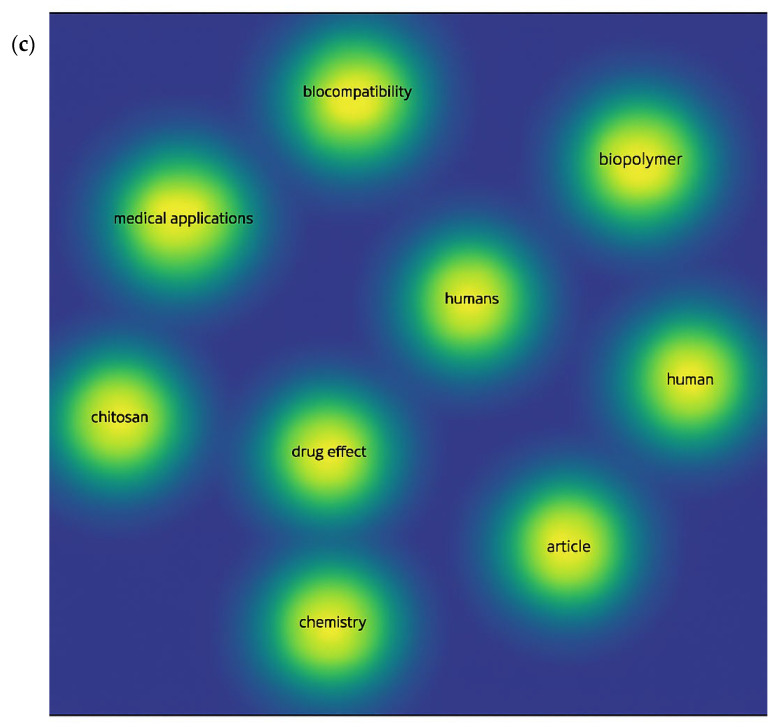
Bibliometric analysis with VOSviewer. (**a**) The co-occurrence network of 74 authors where each node represents a keyword; node size indicates frequency, line thickness reflects co-occurrence strength, and colors denote thematic clusters. The main clusters correspond to the following categories: Green: Chitosan-based nanocomposites and antimicrobial/wound-healing studies. Red: Hydrogels and wound-dressing applications. Cyan: Biomaterials and biodegradable polymers. Purple: Tissue engineering and 3D printing. Yellow: Self-assembly and crosslinking processes. (**b**) A refined analysis of the nine most recurrent terms, highlighting strong connections among medical applications, biocompatibility, biopolymer, and chitosan. These keywords show the highest frequency (occurrences ≥5) and total link strength (TLS), confirming their centrality in current research trends. (**c**) A density visualization of the same network, where color intensity marks the concentration of high-frequency and high-TLS keywords. Hotspots coincide with “biocompatibility”, “medical applications”, and “biopolymers”, visually emphasizing the dominant research focus in the field.

Consistent with the settings reported above (and the terminology in Table 2), we generated a co-occurrence map using author keywords, full counting, and a minimum threshold ≥5; under these parameters, *n* = 74 terms met the threshold (Figure 2a). To complement the interpretation, we include the corresponding density map (Figure 2b), which highlights hotspots of higher frequency and total link strength (TLS) and corroborates the observed clusters. Figure 2c show a density visualization of the same network map (Keywords appear in English as indexed in Scopus.)

#### 2.1.7. Analysis of Bibliometric Data with AI Tools

To complement the PRISMA methodology, an advanced analysis was implemented with generative artificial intelligence (GenAI) to create statistical graphics, data analysis, and interpretations from the bibliographic map exported from Scopus (in .csv format). Also, the results tables associated with each objective were processed with DeepSeek (https://chat.deepseek.com/, accessed on 10 May 2025) and ChatGPT (v5) (https://chatgpt.com/) to identify patterns, correlations, and key trends.

This approach allowed us to optimize the generation of representative graphs, ensuring that each visualization accurately and rigorously reflected the findings reported in the literature. AI tools facilitated the structuring of complex data, the selection of the most appropriate type of graph (e.g., bar charts, bubble maps), and the validation of consistency between source tables and visual representations, thus reinforcing the transparency and reproducibility of the analysis.

## 3. Results

### 3.1. Thermally Conductive Biopolymers in Oncological Treatments

Table 3 summarizes the modification strategies and synthesis methods (e.g., in situ coprecipitation, ionic gelation, ring-opening polymerization (ROP)) and characterization techniques (Fourier transform infrared spectroscopy (FTIR), X-ray diffraction (XRD), scanning electron microscopy (SEM), transmission electron microscopy (TEM), thermogravimetric analysis (TGA), Ultraviolet–Visible Spectroscopy (UV-Vis), sample magnetometry (VSM), Differential Scanning Calorimetry (DSC)) used to study thermally conductive biopolymers.

**Figure 3:**
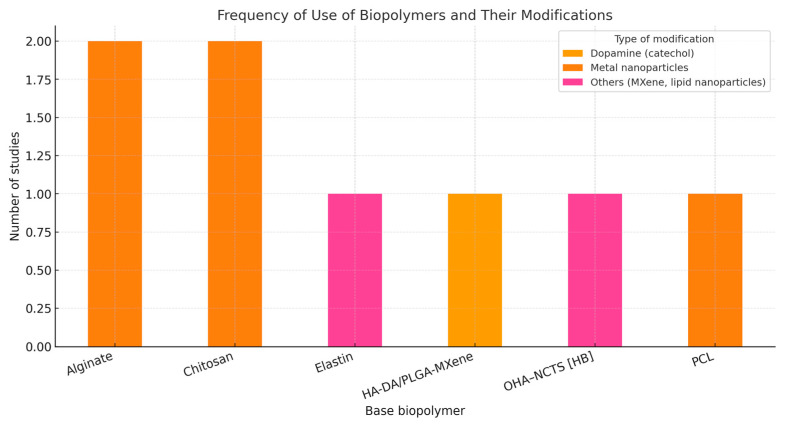
Frequency of use of biopolymers and their modifications.

Figure 3 reveals that alginate and chitosan were the most frequently used base biopolymers, each reported in two studies, primarily modified with dopamine (catechol) to enhance surface functionality and bioadhesion. Elastin, HA-DA/PLGA-MXene, OHA-NCTS[HB], and PCL appeared with lower frequency (one study each), representing alternative formulations incorporating either metal nanoparticles or other modifiers such as MXene and lipid nanoparticles. This distribution indicates a research trend favoring naturally derived polysaccharides functionalized with catechol groups for improved physicochemical and biological performance.

**Figure 4:**
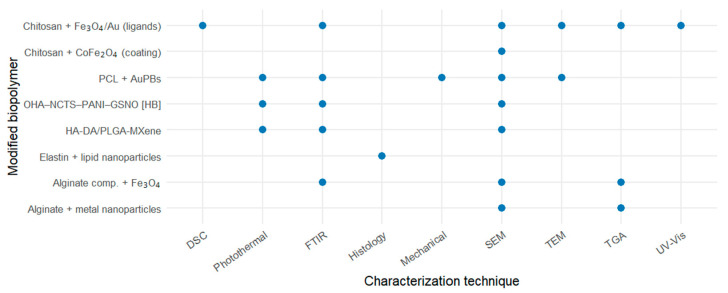
Relationship between modified biopolymers and characterization techniques.

The analysis of Figure 4 demonstrates that the characterization of modified biopolymers commonly involved complementary physicochemical and structural techniques. Fourier transform infrared spectroscopy (FTIR) and scanning electron microscopy (SEM) were the most frequently applied methods, reflecting their relevance in assessing chemical interactions and morphological features, respectively. Thermal analyses (TGA and DSC) and spectroscopic methods (UV–Vis) were also employed to evaluate thermal stability and optical properties. Mechanical and histological assays were less frequent but provided valuable insight into material performance and biocompatibility. Overall, this pattern indicates a multidisciplinary approach to characterizing hybrid biopolymeric systems, integrating structural, thermal, and functional analyses.

**Figure 5:**
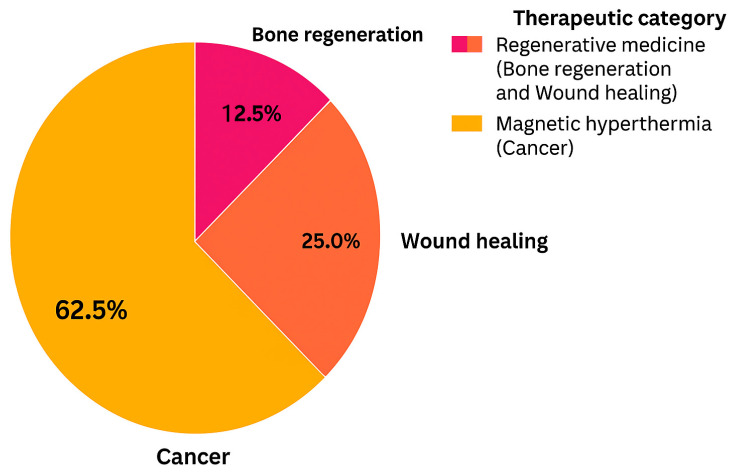
Distribution of biomedical applications grouped into hyperthermia and regenerative medicine categories.

The analysis (Figure 5) revealed that the predominant biomedical application of modified biopolymers corresponds to cancer therapy through magnetic hyperthermia, accounting for 62.5% of the studies reviewed. Regenerative medicine represented the remaining 37.5%, with 25.0% focused on wound healing and 12.5% on bone regeneration. This distribution highlights a strong research emphasis on the development of multifunctional biopolymeric systems for oncological applications, while regenerative approaches remain an important secondary focus aimed at promoting tissue repair and regeneration.

**Figure 6:**
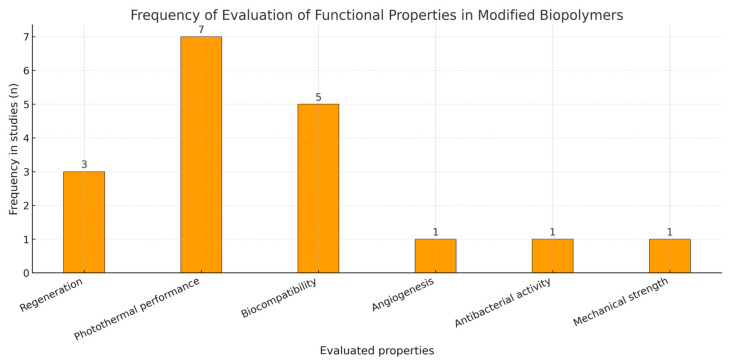
Frequency of evaluation of properties present in thermally conductive biopolymers.

Figure 6 assessment of functional properties reveals that photothermal performance and biocompatibility were the most frequently evaluated parameters, reported in seven and five studies, respectively. Regenerative capacity was analyzed in three studies, while angiogenesis, antibacterial activity, and mechanical strength were each examined only once. This trend indicates a predominant research focus on optimizing photothermal efficiency and biological compatibility, aligning with the growing interest in multifunctional biomaterials for cancer therapy and tissue regeneration.

**Figure 7:**
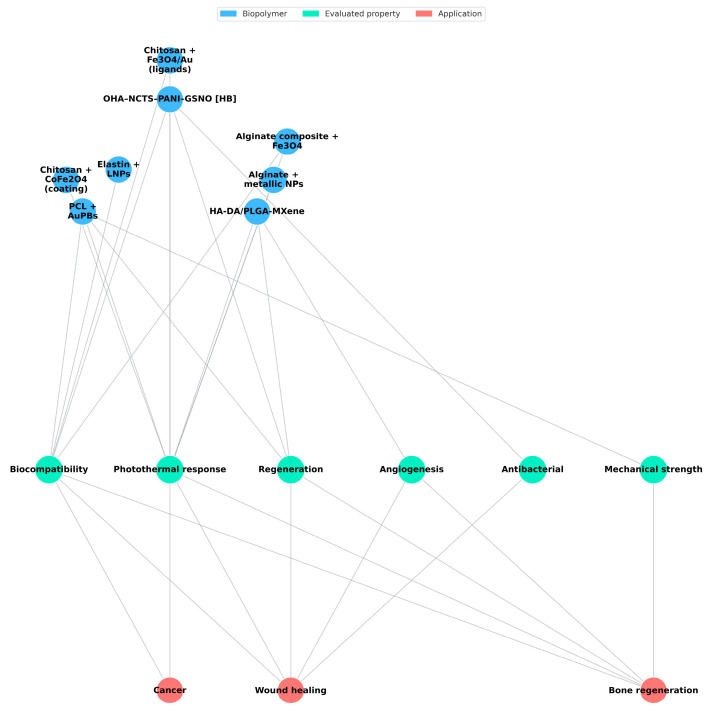
Interrelation between modified biopolymers, evaluated properties, and biomedical applications. Representation of modified biopolymers’ experimentally evaluated properties and biomedical applications.

The network visualization (Figure 7) highlights the interconnectedness between modified biopolymers, their functional properties, and their therapeutic applications. Photothermal response and biocompatibility emerged as central nodes, linking multiple biopolymer systems—such as chitosan- and alginate-based composites—to cancer and wound-healing applications. Regeneration, angiogenesis, and antibacterial activity appeared as secondary connections, primarily associated with regenerative medicine. These interactions reveal that multifunctional composites are being designed to couple thermal and biological performance, particularly targeting cancer therapy while maintaining compatibility with tissue engineering objectives.

### 3.2. Thermally Conductive Biopolymers

Table 4 summarizes conductive biopolymers’ physical (porosity, modulus, thermal conductivity) and chemical (functional groups, crosslinking, degradation) properties.

**Figure 8:**
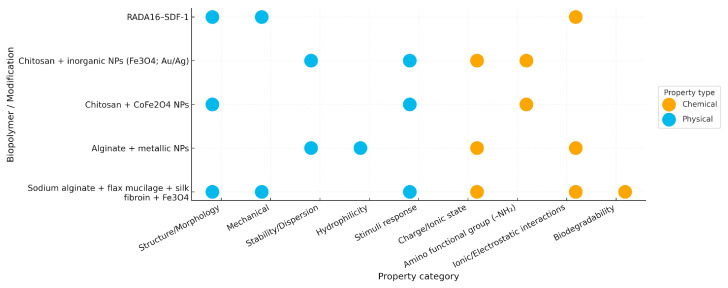
Bubble diagram showing the physical and chemical properties of thermally conductive biopolymers.

Figure 8, the bubble chart, illustrates the distribution of physical and chemical property assessments among different biopolymer modifications. Physical characteristics such as structure/morphology, mechanical stability, and hydrophilicity were the most frequently analyzed, reflecting their importance in determining material performance and dispersion behavior. In contrast, chemical attributes, including ionic and electrostatic interactions, amino functional groups, and biodegradability, were evaluated to understand the influence of surface chemistry on biofunctionality. The balanced focus on both physical and chemical parameters underscores the integrative approach adopted to optimize the physicochemical properties of biopolymer-based composites for biomedical use.

### 3.3. Clinical and Preclinical Studies

Table 5 summarizes the clinical and preclinical studies with different experimental approaches for diverse biomedical applications.

**Figure 9:**
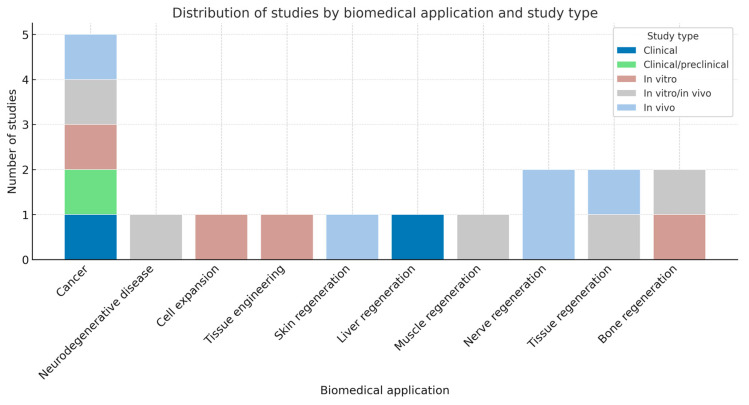
The frequency of studies on thermally conductive biopolymer applications classified according to their experimental model.

The distribution analysis in Figure 9 reveals that cancer-related research accounted for the highest number of studies, encompassing all experimental approaches—from in vitro to in vivo and clinical evaluations—highlighting its prominence in biopolymer-based biomedical investigations. Regenerative medicine applications, including tissue, bone, nerve, and muscle regeneration, were also frequently represented, predominantly at the in vitro or in vivo levels. In contrast, areas such as neurodegenerative disease, cell expansion, and liver regeneration showed limited representation. Overall, the predominance of preclinical and in vivo studies underscores the translational potential of modified biopolymers in therapeutic development, particularly for oncological and regenerative applications.

**Figure 10:**
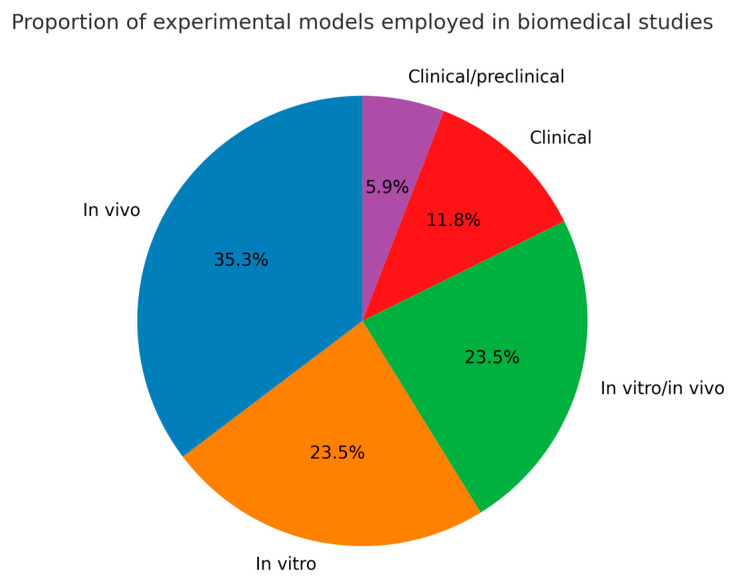
Percentage of applications of thermally conductive biopolymers according to the experimental model.

The analysis revealed (Figure 10) that in vivo models were the most employed, representing 35.3% of the studies, followed by in vitro and combined in vitro/in vivo approaches, each accounting for 23.5%. Clinical studies constituted 11.8% of the total, whereas clinical/preclinical hybrid designs represented only 5.9%. This distribution indicates that preclinical experimentation remains the predominant strategy for evaluating modified biopolymers, reflecting an emphasis on translational validation before clinical application.

**Figure 11:**
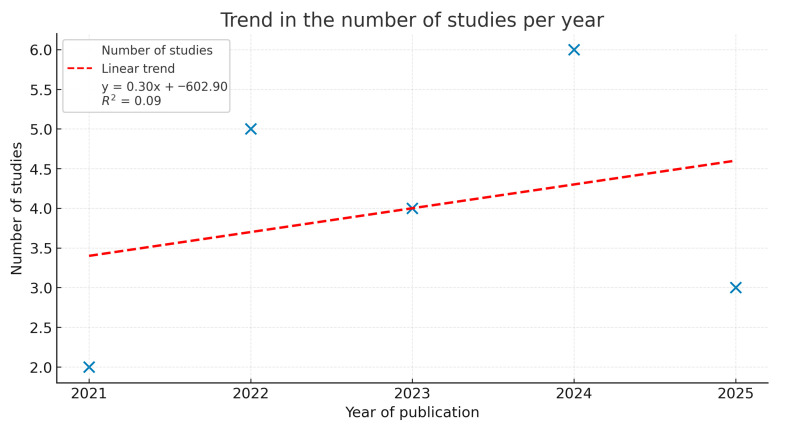
The colored dotted line reflects the growth of research. The value of R^2^ = 0.16 explains a weak correlation.

Figure 11 shows temporal distribution of publications shows a slight upward trend in the number of studies on modified biopolymers between 2021 and 2025, as indicated by the positive slope of the linear regression (y = 0.30x − 602.90; R^2^ = 0.09). Although the coefficient of determination suggests a weak correlation, the data reflect a gradual increase in research activity, with a peak observed in 2024. This pattern denotes growing scientific interest in the field, likely driven by advancements in nanocomposite design and biomedical applications.

Table 6 compiled the quantitative results of the efficiency and safety parameters.

**Figure 12:**
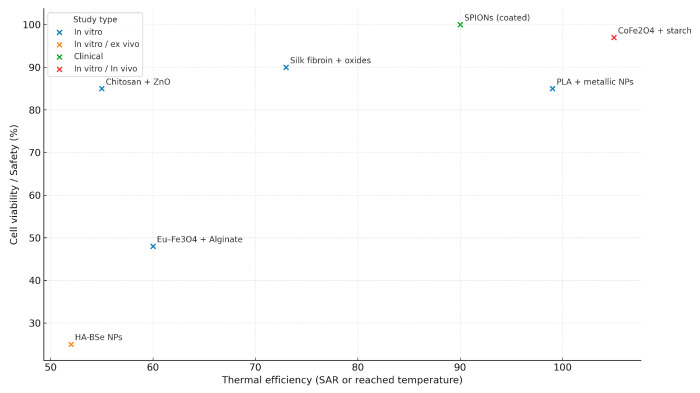
The scatter plot represents the relationship between thermal efficiency and safety, describing the type of biopolymer applied and the type of study.

Figure 12 shows relationships between thermal efficiency and biocompatibility, revealing that most modified biopolymer systems exhibited high cell viability (>80%) while maintaining effective thermal responses. CoFe_2_O_4_–starch and coated SPIONs demonstrated optimal performance, combining high thermal efficiency (>90) with near-complete cellular safety. In contrast, HA–BSe nanoparticles showed reduced biocompatibility despite moderate heating capacity, indicating potential cytotoxicity. Overall, these results suggest that surface functionalization strategies, such as polymer coating and biocompatible matrix incorporation, enhance both safety and thermal performance, supporting their potential for clinical translation in hyperthermia-based therapies.

**Figure 13:**
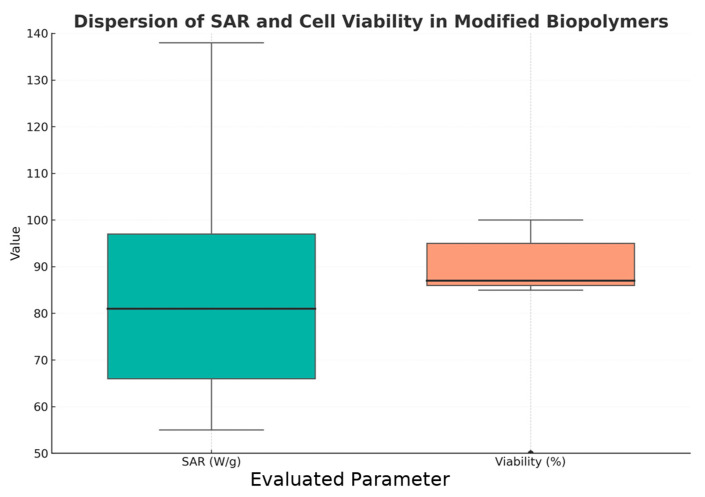
The SAR parameter shows greater dispersion than cell viability, with greater variability in thermal efficiency. Cell viability shows higher values, which are understood as indicating efficient biocompatibility. The graph shows the interquartile range, the median, and the maximum and non-atypical values.

The boxplot analysis shows (Figure 13) that SAR values exhibited a wider dispersion (ranging from approximately 50 to 140 W/g) compared to cell viability, which remained consistently high (85–100%). The median SAR (~80 W/g) indicates variable but generally efficient photothermal or magnetothermal responses among the evaluated materials. In contrast, the narrow variability in cell viability reflects the overall cytocompatibility of the modified biopolymers, suggesting that most formulations achieve a balance between therapeutic heating capacity and biological safety.

Table 7 integrates the relationship between concentration, applied dose, and treatment.

**Figure 14:**
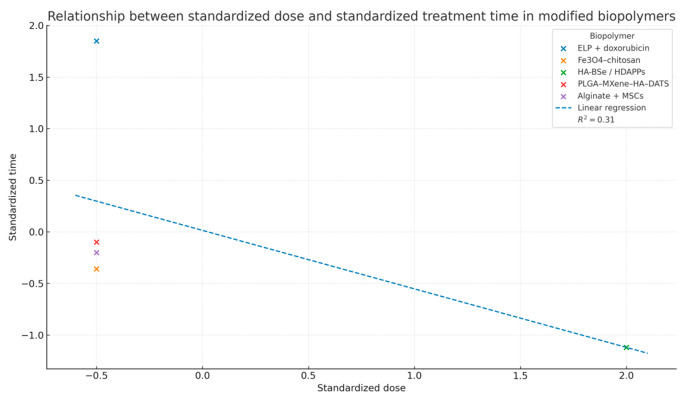
Relationship between standardized dose and standardized treatment time in modified biopolymers.

Figure 14 establishes that a negative correlation (R^2^ = 0.31) was observed between standardized dose and treatment time across different modified biopolymers, indicating that higher administered doses are generally associated with shorter treatment durations. Among the analyzed systems, ELP–doxorubicin and Fe_3_O_4_–chitosan composites exhibited the most pronounced responses, suggesting efficient therapeutic performance at reduced exposure times. This trend reflects a dose-dependent efficacy pattern, highlighting the potential of optimized biopolymer formulations to achieve therapeutic outcomes with minimized treatment intervals.

**Figure 15:**
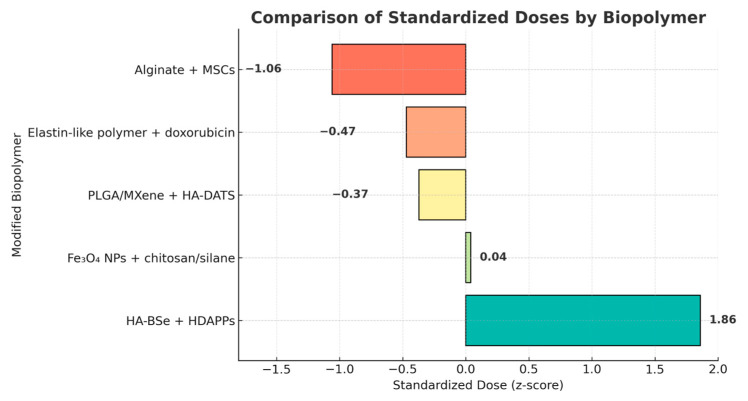
The standardized data (z-score) is used to identify which doses are above and below average.

The comparative analysis (Figure 15) of standardized doses (expressed as z-scores) revealed notable variability among the evaluated biopolymeric systems. The alginate–MSC composite exhibited the highest relative dose (z = 1.86), indicating a substantially greater therapeutic load compared to other formulations. In contrast, HA–BSe/HDAPPs, Fe_3_O_4_–chitosan/silane, and PLGA–MXene/HA–DATS presented negative z-scores, suggesting lower administered concentrations. The elastin-like polymer–doxorubicin system displayed a value near the mean (z = –0.04). These findings reflect diverse dosing strategies influenced by material composition and application type, underscoring the adaptability of modified biopolymers to specific therapeutic requirements.

### 3.4. Biomedical Applications 

Table 8 summarizes the specific applications of thermally conductive biopolymers in regenerative medicine.

**Figure 16:**
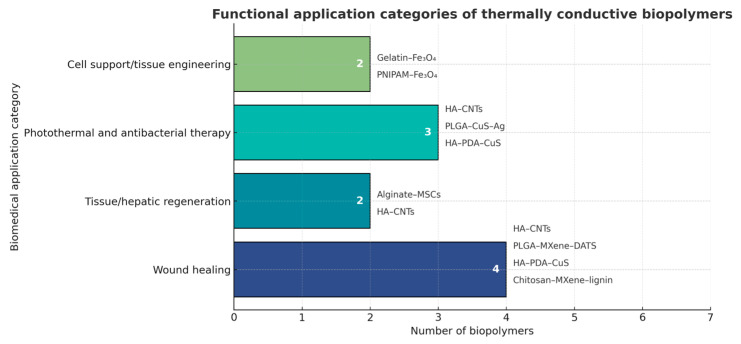
Applications of thermally conductive biopolymers in regenerative medicine.

Figure 16 shows analysis revealed that wound healing represented the most prevalent functional category, encompassing four distinct thermally conductive biopolymer systems, including chitosan–MXene–lignin and HA–CNTs. Photothermal and antibacterial therapies followed, represented by three materials, while cell support/tissue engineering and tissue or hepatic regeneration each accounted for two systems. This distribution indicates a predominant focus on wound healing and infection control applications, reflecting the growing interest in harnessing the dual regenerative and antimicrobial potential of thermally active biopolymer composites.

The distribution of biomedical applications across biopolymer functional groups revealed that scaffolds and hybrid systems were the most versatile, each encompassing multiple therapeutic categories. Scaffolds were primarily associated with wound healing, regeneration, and thermotherapy-related applications, highlighting their multifunctionality and structural adaptability. Hybrid systems integrated a wider range of functions, including photothermal, antibacterial, and regenerative activities, reflecting their potential for combinatorial therapies. In contrast, hydrogels and platforms were less represented, focusing mainly on hepatic regeneration and antibacterial purposes. These findings emphasize the versatility of composite scaffold and hybrid architectures in addressing complex biomedical challenges.

Table 9 provides a comparative synthesis of the in vitro and in vivo applications of thermally conductive biopolymers. For each study, it reports the biopolymer matrix/composition (e.g., chitosan, alginate, GelMA, and PLA/PLGA composites with Au, Fe_3_O_4_/CoFe_2_O_4_, CuS, Ag, or MXenes), the application and treatment modality (magnetic or photothermal hyperthermia, controlled drug release, tissue regeneration), the study type/model, the modification/fabrication method (functionalization, crosslinking, 3D printing, core–shell systems), and thermal performance (e.g., SAR/ILP, target temperatures, CEM43 where available).

It also summarizes outcome highlights (efficacy/biocompatibility) and the clinical status (preclinical vs. early clinical) to enable side-by-side appraisal of performance and translational readiness. Notably, the table highlights materials demonstrating localized, controllable heating with acceptable safety and flags entries with clinical evaluation, thereby clarifying near-term candidates and remaining evidence gaps.

## 4. Discussion

In agreement with the established aims of this project, a brief discussion of the principal findings supported by the reviewed literature is presented.

(a) Biopolymer modification strategies

Firstly, it was found that biopolymers such as cellulose–graphene nanocomposites and chitosan–graphene oxide blends [43] perform well in oncological treatments. These materials can generate heat when exposed to light or magnetic fields, which is essential in therapies like hyperthermia, where the goal is to increase tumor tissue temperature to induce cancer cell death [2]. Additionally, Djoudi et al. [44] showed that hyaluronic acid, when forming conductive hydrogels, can also be helpful for localized treatments in the nervous system.

Other studies, e.g., Zhang et al. [45], indicated that incorporating carbon nanotubes into starch significantly improves thermal conductivity, enabling more precise treatment of affected tissues. Similarly, Hazarika and Borah [9] employed europium-doped iron oxide nanoparticles within a chitosan matrix. They confirmed their ability to generate heat without damaging healthy tissues, highlighting the importance of combining thermal conductivity with biocompatibility. Along the same lines, Adorinni et al. [46] emphasized the value of hydrogels enriched with carbon-based nanomaterials, such as graphene and nanotubes, for controlled drug release triggered by temperature increases applicable in cancer treatment and tissue regeneration.

Although these benefits were identified, several limitations were also identified. For example, Mohammad et al. [10] noted that while these materials can induce cancer cell death, they may also affect healthy cells by generating thermal stress that activates defense mechanisms such as heat shock protein 70 (HSP70). Similarly, Fadeel et al. [47] cautioned that prolonged or inappropriate use of carbon nanomaterials could compromise cellular health, reinforcing the need for precise dosage and application protocols. The limitations include (i) potential off-target heating of healthy cells leading to thermal stress; (ii) induction of heat shock responses (notably HSP70) that may diminish antitumor efficacy; (iii) need for improved thermal selectivity and spatial targeting; (iv) risks associated with prolonged or inappropriate exposure to carbon nanomaterials; (v) strict dependence on dosing and exposure protocols.

(b) Hyperthermia applications

Concerning the safety and compatibility of these biopolymers (see Table 3, Table 4, Table 5, Table 6, Table 7 and Table 8), Fiorentini et al. [4] demonstrated that temperature-sensitive materials used in regional hyperthermia improve drug distribution within tumors while minimizing damage to surrounding healthy tissues. Yang et al. [48] also observed that graphene-based nanomaterials are compatible with nervous tissue, making them suitable for therapeutic use without causing inflammation. Dragojevic, Hall, and Raucher [3] introduced an innovative approach using elastin-like polypeptides to deliver doxorubicin, a widely used chemotherapy drug. These biopolymers enable the drug to accumulate in tumors through mild hyperthermia, reducing adverse effects such as cardiac toxicity.

Biocompatibility was also highlighted in studies by Teijeiro-Valiño et al. [49] and Aslibeiki et al. [50], where materials such as gelatin and modified starch showed no evidence of tissue rejection. This is important for avoiding undesirable immune responses. Furthermore, Radhouani et al. [51] added that the ability to tailor the properties of biopolymers makes it possible to design materials for various medical applications without causing adverse effects.

(c) Clinical and preclinical evidence

In regenerative medicine, Jin et al. [52] noted that polysaccharides can be used to construct scaffolds and biosensors that foster favorable cell growth and differentiation environments. Darie-Niță and Frąckowiak [53] also highlighted the value of designing sustainable hybrid polymers that perform well and reduce environmental impact.

Haririan and Ysefnejad [54] studied the use of conductive hydrogels for wound healing and observed that they help stimulate collagen production and tissue regeneration. However, as Mayekar and Auras et al. [55] pointed out, valuable materials such as polylactic acid (PLA) may trigger inflammation if their degradation is not adequately controlled. These biopolymers still need surface and formulation refinements to prevent undesired side effects.

Among the emerging research lines are innovative materials such as G-quadruplex hydrogels and chitosan-functionalized nanobubbles [56], which exhibit properties like self-healing and targeted drug delivery. These technologies could make a significant difference in treating complex diseases and developing more effective regenerative therapies.

In summary, the findings of this review confirm that thermally conductive biopolymers hold great promise in oncology and regenerative medicine. Their ability to generate localized heat, biocompatibility, and tunable properties positions them as a compelling option for developing innovative treatments.

The evidence, supported by Table 3, Table 4 and Table 5, validates the key concepts studied and opens new opportunities for advancing safer and more effective biomaterials. The compiled evidence indicates that thermally conductive biopolymers—chitin/chitosan, gelatin/collagen, GelMA, PNIPAM, hyaluronic acid, and PLA/PLGA—functionalized with photothermal or magnetic nanoagents (Au, Fe_2_O_3_/Fe_3_O_4_/CoFe_2_O_4_, CuS/Ag, MXene–Nb_2_C) are consolidating as versatile platforms for localized hyperthermia and controlled drug delivery in oncology, with applications dominated by oncologic and tissue-repair indications (see Figure 3).

Photothermal behavior emerges among the most frequently assessed properties (see Figure 4). Recent output is shifting toward in vivo and combined models (see Figure 5). In terms of antitumor efficacy, significant tumor-volume reductions and good tolerability have been documented for elastin-like systems with doxorubicin and for modulated electro-hyperthermia approaches, with no added cardiotoxicity when thermal dosimetry is properly controlled [3,4].

Biopolymers also serve as delivery platforms: chitosan and gelatin have carried gold and iron oxide nanoparticles for targeted release and hyperthermia with localized ablation of tumor cells [6,57], whereas hyaluronic-acid hydrogels with Fe_2_O_3_ or polydopamine–PEG have shown tumor destruction under NIR irradiation and benefits in breast cancer [45,52].

This performance is underpinned by functional properties—thermal stability, viscoelasticity, thermal transitions, and π–π interactions—linked to intermolecular interactions (hydrogen bonding, β-sheet domains) that strengthen structural integrity and therapeutic action, consistent with Lima-Sousa and Correia [55] and Radinekiyan et al. [31]; experimental verification recurrently relies on DSC/TGA, FTIR, SEM, and mechanical and histological assays [31], (see Figure 2). Even so, there is an efficiency–cytotoxicity trade-off: combinations with Eu-Fe_3_O_4_ or HA-BSe reach temperatures of ≈50 °C with decreased viability, whereas other formulations (PLA with metallic nanoparticles; CoFe_2_O_4_–starch) achieve SAR ≥ 100 W/g while maintaining viability ≥85% [9,11,33] (see Figure 6 and Figure 7).

Hence, there is a need to standardize parameters (H·f product, laser wavelength/power or AMF, exposure time) and SAR calculation and reporting criteria [58]. Regarding safety and biocompatibility, polymer coatings and core–shell architectures limit ion release and support tolerability; for example, chitosan/aminosilane-coated SPIONs induced apoptosis in 90% of the treated tumor volume without iron release, and functionalized iron oxide nanoparticles showed antitumor effects without notable toxicity [2,59].

**Figure 17:**
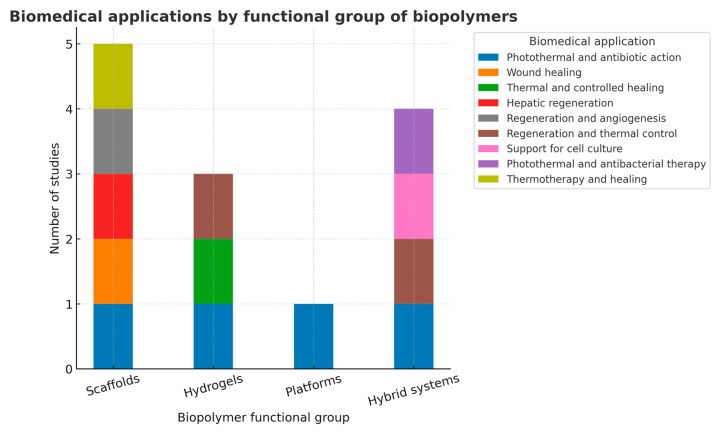
Classification of thermally conductive biopolymers according to functional application.

In comparison, evidence for tissue regeneration is consistent: hybrid systems and scaffolds—often based on PLGA/MXene-DATS, HA-CNTs, and related composites—integrate structural support plus thermal conduction plus release to enhance angiogenesis and wound healing [52,60] (see Figure 16 and Figure 17).

Additional therapeutic scenarios include liver regeneration via alginate with MSCs and bone regeneration using gelatin + iron oxide nanoparticles, supported by preclinical results and by nano-reinforced biopolymeric scaffolds [39]; emerging applications in neuroregeneration and neurodegenerative diseases likewise call for new functionalization strategies [61].

Methodologically, the concentration–regimen relationship and the inverse dose–time correlation distinguishes localized therapies from regenerative approaches [62] (see Figure 12 and Figure 13). The study-type landscape shows (Figure 8) growing reliance on in vivo and combined models, aligning with calls to validate in more complex experimental systems [63].

### 4.1. Comparative Analysis Across Thermally Conductive Systems

Comparative analysis reveals distinct trade-offs between hydrogels, nanocomposites, metallic, and carbon-based agents that determine their biomedical suitability. Hydrogel matrices (e.g., GelMA, OHA/polyaniline, hyaluronic acid–based scaffolds) offer superior biocompatibility, flexibility, and nutrient diffusion, making them ideal for soft-tissue and regenerative applications where moderate, uniform heating (≈41–42 °C) promotes angiogenesis and healing. In contrast, nanocomposites, and metallic fillers such as Fe_3_O_4_, CoFe_2_O_4_, and Au nanoparticles deliver higher thermal conductivity and rapid heat generation but demand stringent control to avoid off-target heating and oxidative stress. Meanwhile, carbon-based materials (graphene, CNTs) bridge these categories, combining mechanical reinforcement and photothermal responsiveness with adjustable conductivity.

However, they raise immunogenicity and long-term clearance concerns that limit clinical translation. Therefore, the most effective strategies increasingly involve hybrid systems, for example, integrating magnetic or plasmonic nanostructures into biopolymer hydrogels balancing safety, precision, and durability for oncology and regenerative medicine applications. These findings emphasize that future designs should adopt context-specific hybridization, aligning material selection with thermal dose, tissue type, and clinical endpoint to maximize therapeutic efficacy.

### 4.2. Risks and Translation Constraints (Toxicity, Scalability, Regulation, and Stability)

Despite encouraging efficacy signals, nano-enabled biopolymers face non-trivial risks that remain incompletely characterized. First, toxicity can arise from off-target heating and stress responses when thermal dose is not tightly controlled; metal or carbonaceous fillers may contribute to ion release, ROS, or chronic tissue persistence, which are under-reported beyond short-term models [64,65,66,67].

Second, long-term stability is seldom quantified with in vivo degradation kinetics (mass loss, ion/particle clearance), thermo-mechanical fatigue under repeated heating, or shelf-life/sterility (endotoxin) dossiers—factors essential for clinical reliability [37]. 

Third, scalability poses hurdles: reproducible functionalization/dispersion, batch-to-batch control of size/ζ-potential/crosslink density, and transition to cGMP processes for 3D-printed or injectable formats remain weakly documented [68].

Finally, regulatory progress requires standardized dosimetry and reporting—e.g., SAR/ILP with frequency/amplitude for magnetic hyperthermia and full optical parameters for photothermal studies [69], plus comprehensive ISO 10993 [70] biocompatibility panels with immunoprobing under current device/combination-product frameworks [71,72].

Addressing these gaps already reflected by the predominance of in vitro/small-animal data and heterogeneous reporting in the current literature should be prioritized to convert laboratory performance into durable, safe, and scalable clinical products [73,74,75,76].

### 4.3. Advantages of Thermally Conductive Biopolymers over Other Polymers in Regenerative Medicine and Oncology

Thermally conductive biopolymers offer several advantages compared with conventional polymeric materials in regenerative medicine and oncology. Their primary benefit lies in their ability to combine precise thermal management with intrinsic biocompatibility and biodegradability, enabling localized hyperthermia without damaging surrounding healthy tissue. Unlike traditional polymers, which are thermally insulating, biopolymers such as chitosan, alginate, hyaluronic acid, and PLGA can be functionalized with magnetic or photothermal nanostructures (e.g., Fe_3_O_4_, Au, CoFe_2_O_4_, MXenes) to efficiently convert external energy sources into controlled heat. This property supports targeted tumor ablation, controlled drug release, and enhanced angiogenesis and osteogenesis in tissue regeneration.

Moreover, their natural origin allows cell adhesion, nutrient diffusion, and integration with host tissues, key for wound healing, bone formation, and neural regeneration. The dual functionality, thermal modulation, and biological compatibility, positions these materials as superior alternatives to synthetic polymers that often require external coatings or toxic crosslinkers to achieve similar performance. In oncology, this combination translates into selective hyperthermia, reduced systemic toxicity, and synergistic action with chemotherapy or radiotherapy, while in regenerative medicine, it enables non-invasive stimulation of repair processes under mild hyperthermic conditions (≈41–42 °C), fostering vascularized and functional tissue recovery.

### 4.4. Hurdles for Thermally Conductive Biopolymers in Clinical Applications

Despite promising laboratory results, several critical hurdles limit the clinical translation of thermally conductive biopolymers. Foremost are issues related to safety, reproducibility, and regulatory compliance. Many systems rely on metallic or carbonaceous fillers whose long-term biostability, degradation kinetics, and immunogenicity remain incompletely characterized. Uncontrolled heat generation can cause off-target thermal injury, oxidative stress, or inflammation, especially when dosimetry is not standardized. Moreover, batch-to-batch variability in nanoparticle dispersion, crosslinking density, and surface chemistry complicates scale-up and hinders compliance with Good Manufacturing Practice (cGMP) standards. Another major limitation is the lack of harmonized dosimetry and thermometry protocols—such as standardized SAR/ILP reporting and optical power calibration—which impedes inter-study comparability and regulatory evaluation. Clinically, evidence is still dominated by in vitro and small-animal models, with few long-term or large-scale trials validating biocompatibility and therapeutic durability in complex tissue environments. Finally, clearance and biodegradation pathways for nano-enabled composites are poorly defined, posing potential accumulation and toxicity risks. Overcoming these barriers will require standardized reporting, longitudinal in vivo safety studies, scalable fabrication protocols, and integrated regulatory frameworks to bridge the gap between preclinical innovation and reliable human applications.

### 4.5. Roadmap for Interdisciplinary Collaboration

This roadmap serves as a strategic framework to integrate our aims and ensure that our efforts are aligned and mutually reinforced, maximizing the impact of our research.

**Figure 18:**
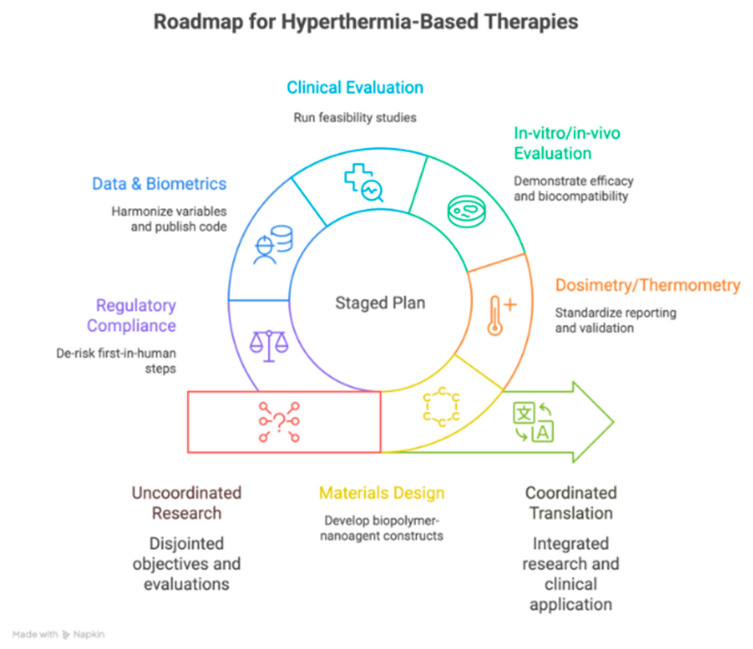
Roadmap. Designed by NapKin AI https://app.napkin.ai/ (accessed on 15 October 2025).

Figure 18 outlines a strategic roadmap for interdisciplinary collaboration, aligning with the objectives of hyperthermia-based therapies, clinical/preclinical evidence appraisal, and regenerative medicine applications. It presents a staged plan coordinating materials design, dosimetry/thermometry, in vitro/in vivo/clinical evaluation, and translational efforts. The roadmap leverages PRISMA synthesis and VOSviewer mapping to prioritize key themes and partnerships, focusing on materials like chitosan/alginate/GelMA and nanoparticle platforms such as Au, Fe_2_O_3_/Fe_3_O_4_/CoFe_2_O_4_, CuS, and MXenes (Nb_2_C), within the dual domains of oncology and regeneration.

## 5. Conclusions

Through this review, we conclude that thermally conductive biopolymers (e.g., chitosan, alginate, GelMA, PNIPAM, and PLA/PLGA) functionalized with magnetic or photothermal nanoagents (e.g., Fe_3_O_4_/CoFe_2_O_4_, Au, CuS, MXenes) show strong potential for localized hyperthermia and regenerative medicine. However, three critical gaps must be addressed to enable clinical translation:

(i) Long-term biocompatibility and safety. There is a paucity of longitudinal in vivo data on degradation/clearance (mass loss, ion/particle release), immunogenicity, oxidative stress, and tissue remodeling under repeated thermal cycles.
(ii) Preclinical to clinical translation. In vitro and small-animal studies dominate evidence; progress requires larger animal models, quantitative pharmacokinetics/retention for carriers, and endpoints aligned to clinical realities (tumor heterogeneity, perfusion-driven heat sinks, complex tissue microenvironments).
(iii) Standardized protocols and reporting. We advocate harmonized dosimetry/thermometry—including SAR/ILP with field parameters for magnetic hyperthermia, full optical descriptors for photothermal therapy, and CEM43—alongside consistent ISO-10993 [70]—aligned biocompatibility panels with immune profiling and reproducible inter-lab procedures.

Collectively, cGMP-ready manufacturing, longitudinal safety dossiers, and comparative head-to-head evaluations of magnetic vs. photothermal platforms will be pivotal to harness observed efficacy with minimal toxicity and scalable clinical deployment.

### Limitations and Future Directions

This review covers literature advances from 2020 to 2025 concerning high-impact studies in magnetic and photothermal hyperthermia and regenerative platforms, ensuring contemporaneous scope and citation currency. Nevertheless, the literature is heavily weighted toward in vitro assays and small-animal models, with scarce clinical studies and limited external validation.

Additionally, to bridge the mentioned gaps, we recommend a minimal, field-agnostic reporting set (materials/nanoagents descriptors; full dosimetry/thermometry; ISO-10993-aligned immune panels), multi-site large-animal validation with indication-specific endpoints and extended follow-up, and closed-loop, image-guided thermal control tailored to target tissues. Open datasets and analysis scripts (including bibliometrics and extracted tables) should accompany future studies to enable reproducibility and meta-analysis, accelerating the path from bench performance to durable, safe, and scalable clinical products.

This systematic review was registered in PROSPERO as CRD420251165632 [77].

## Figures and Tables

**Table 1:** Boolean operators for looking at documents on thermally conductive biopolymers.

Topic	Keywords	Boolean Operators	Advance Search
Material	Biopolymer, conductive polymer, conjugated polymer, thermal conductivity, nanocomposite	AND, OR	(“biopolymer” AND “thermal conductivity”), (“conjugated polymer” OR “nanocomposite”), (biopolymers AND medical AND oncology)
Properties	Biocompatible, biodegradable, thermal stability, processability	AND, OR	(“biocompatible” AND “biodegradable”), (“thermal stability” AND “processability”)
Mechanisms of action	Local heating, hyperthermia, controlled drug release, tissue regeneration	AND, OR	(“local heating” AND tumor), (hyperthermia AND “tissue regeneration”)
Medical applications	Oncology, tumor, cancer, regenerative medicine, tissue, wound	AND	(“oncology” AND biopolymer), (tumor AND “local heating”), (“tissue regeneration” AND “conductive biopolymers”), (biopolymer AND radiotherapy), (“biopolymers” AND medicine AND oncology)
Specific applications	hyperthermic therapy, controlled drug release, tissue engineering	AND	(“hyperthermic therapy” AND tumor), (“controlled release” AND drug AND biopolymer), (tissue AND engineering AND “biopolymers” AND immunogenicity), (“thermally conductive biopolymers” AND “combination therapy”), ((“polylactic acid” OR “PLA” OR “polylactic acid polymer”) AND (“medical application*” OR “biomedical application*”))
Material properties	thermal conductivity, biocompatibility, thermal stability, processability, mechanical properties	AND, OR	(“thermal conductivity” AND biocompatibility), (“mechanical properties” AND “conductive polymer”), (biopolymer AND (conductivity OR thermal) AND medicine), (“biopolymers” AND chemical AND properties AND biomedicine)

The symbol (*) represents a wildcard to help in the search when a word has multiple spelling variations.

**Table 2:** Analysis types and analysis units available in VOSviewer.

Analysis Type	Analysis Unit
Co-authorship	Authors
	Organizations
	Countries
Co-occurrence	Keywords
	Author Keywords
	Index Keywords
Citation	Documents
	Sources
	Authors
	Organizations
	Countries
Bibliographic coupling	Documents
	Sources
	Authors
	Organizations
	Countries
Co-citation	Cited references
	Cited sources
	Cited authors

**Table 3:** Modification strategies, synthesis methods, and characterization techniques for thermally conductive biopolymers in hyperthermia therapies and regenerative medicine.

Ref.	Base Biopolymer and Modification	Synthesis Method	Characterization Techniques	Evaluated Properties	Application
[28]	HA-DA/PLGA-MXene	3D printing and photocrosslinking	SEM, FTIR, XRD, angiogenic and osteogenic assay	Angiogenesis–osteogenesis, photothermal performance	Vascularized bone regeneration
[20]	Oxidized hyaluronic acid (OHA) + N-carboxyl chitosan (N-CTS) + polyaniline (PANI) + S-nitrosoglutathione (GSNO).	Dynamic copolymerization/crosslinking via Schiff-base bonds.	SEM, FTIR; assessment of photothermal response under NIR	In vitro biocompatibility (L929 and HUVEC > 90% viability); self-healing; conductivity/photothermal effect; sustained NO release; antibacterial activity	Diabetic wound healing (murine model)
[19]	PCL (polycaprolactone) + AuPBs (gold plasmonic blackbodies) → photothermal composite scaffold	3D printing of PCL–AuPB composite	SEM, TEM, FTIR, NIR	Photothermal response (mild hyperthermia, optimal 39–41 °C), mechanical strength, and cell viability	Bone tissue engineering
[3]	Elastin, drug payload with lipid nanoparticles	Molecular self-assembly	In vivo assays, histology	Viability, tumor inhibition, cardioprotection	Breast cancer, targeted drug delivery
[29]	Alginate + metallic nanoparticles	Ionotropic gelation	SEM, thermal analysis, biological tests	Thermal properties, controlled release	Localized oncologic therapies
[6]	Chitosan and derivatives (CMCS/TMC, thiolated); CS–Fe_3_O_4_/Au nanocomposites; ligands (PEG, folic acid)	Ionic gelation (TPP), coprecipitation, emulsification, self-assembly, conjugation.	SEM, UV–Vis spectroscopy FTIR, ^1^H NMR, SEM/TEM, DLS/ζ-potential, XRD, UV–Vis, DSC/TGA; VSM (if Fe_3_O_4_)	Biocompatibility; size/ζ; encapsulation efficiency (EE%) and release; stability; photothermal/magnetic response	Drug/gene delivery; adjuvant hyperthermia; diagnostics/theranostics
[30]	Chitosan-coated CoFe_2_O_4_ nanoparticles	Coprecipitation of CoFe_2_O_4_ + chitosan coating	XRD (Rietveld), SEM, SQUID, heating tests under AMF	Spinel structure confirmed; ferrimagnetic behavior; effective thermal response	In vitro magnetic hyperthermia (KAIMRC2 breast cancer cells)
[31]	Sodium alginate + flaxseed mucilage + silk fibroin; Fe_3_O_4_ nanocomposite	Extraction of mucilage and fibroin (degumming/sericin removal) mix with alginate ionic gelation (CaCl_2_) → in situ coprecipitation of Fe^2+^/Fe^3+^ with NH_4_OH (porous matrix)	FTIR, XRD, SEM, TGA, VSM	High biocompatibility; marked antitumor activity; effective hyperthermia response; good Fe_3_O_4_ dispersion/stability in porous, flexible matrix	In vitro magnetic hyperthermia and anticancer

**Table 4:** Biopolymers physical and chemical properties.

Ref.	Biopolymer/ Modification	Physical Properties	Chemical Properties
[29]	Alginate + metallic nanoparticles	Hydrophilic nature; structural stability	Anionic surface; electrostatic interactions
[32]	RADA16–SDF-1	Nanofibrillar hydrogel; Young’s modulus ≈ 3.21 kPa (like neural tissue)	Amide bonds; ionic interactions
[6]	Chitosan + inorganic nanoparticles (Fe_3_O_4_; Au/Ag)	Colloidal stability/dispersion; thermal response under external stimulus	Amino (–NH_2_) and hydroxyl (–OH) groups; positively charged in acidic media
[31]	Sodium alginate + flaxseed mucilage + silk fibroin; incorporation of Fe_3_O_4_ NPs	Porous, flexible matrix; enhanced mechanical properties; heating under AMF	Negative charge (–COO^−^); –OH/–COO^−^ and amide groups; ionic crosslinking with Ca^2+^; anchoring to Fe_3_O_4_ (–COO^−^↔Fe); biodegradable
[30]	Chitosan functionalized with CoFe_2_O_4_ NPs	Spinel structure; paramagnetic behavior; ability to generate heat under AMF	–NH_2_ and –OH groups (high reactivity)

**Table 5:** Clinical and preclinical studies of biopolymers with biomedical applications.

Ref.	Study Type	Biopolymer/Modification	Experimental Model	Biomedical Application
[3]	In vitro/In vivo	Elastin-like polypeptide (ELP), cell-penetrating peptide SynB1, pH (Low) Insertion Peptide (pHLIP), and doxorubicin (DOX). (ELP + SynB1 + pHLIp + doxorubicin)	4T1 cells and mice	Breast cancer—controlled chemotherapy
[10]	In vitro/In vivo	Cu–Mn_3_O_4_–TMC + 5-Fu	MCF-10 cells and mice	Cancer—chemo-phototherapy
[9]	In vitro	Eu–Fe_3_O_4_ + sodium alginate	HeLa cells	Localized hyperthermia
[11]	In vitro/ex vivo	HA-BSe NPs, HDAPPs	CT26 cells, 3D organoids	Colorectal cancer
[33]	In vitro	Chitosan + collagen + hydroxyapatite	Mesenchymal stem cells	Tissue engineering
[8]	In vitro	IONFs in temperature-sensitive liposomes	A549 cells	Human lung adenocarcinoma
[4]	Clinical	Modulated electro-hyperthermia (mEHT)	217 pancreatic cancer patients	Hyperthermia + chemotherapy
[2]	Clinical/Preclinical	SPIONs coated with chitosan and aminosilane	Phase I [14], Phase II [31] GBM patients	Cancer—magnetic hyperthermia
[34]	In vitro/In vivo	CoFe_2_O_4_ + starch and gum	Cells and mice	Hyperthermia—thermal efficiency and compatibility
[18]	In vitro/In vivo	PLGA + β-TCP + MXene (Nb_2_C)	HUVECs/animal model	Vascularized bone regeneration
[35]	In vitro/In vivo	FPCP (Pluronic F127 + poly (citric acid) + polypyrrole)	C2C12 cells/mice	Muscle regeneration
[19]	In vitro	PCL + AuPB	Bone cells	Photothermal bone regeneration
[28]	In vivo	PLGA-MXene + HA-DATS hydrogel	Mice with wounds	Tissue regeneration
[36]	In vivo	MpGel (graphene + polydopamine + mupirocin)	Mice with diabetic ulcers	Wound healing, nerve regeneration
[37]	In vivo	GeIDA + PgO + mupirocin	Infected burns	Skin regeneration and antimicrobial
[38]	In vivo	CMCs + EGCG + Pt-PVP	Mice	Antioxidant, angiogenesis, anti-inflammatory
[39]	Clinical	Alginate + MSCs	Patients with cirrhosis	Liver regeneration
[40]	In vitro/In vivo	CMC + gold nanoparticles + tannic acid	NSCs/induced Parkinson’s	Therapy for neurodegenerative disease
[41]	In vitro	Alginate + gelatin	Hematopoietic stem cells	Cell expansion for transplantation
[42]	In vivo	G/CST + DPSCs	Rabbit facial nerve	Peripheral nerve regeneration

**Table 6:** Quantitative results for efficiency and safety parameters.

Ref.	Study	Modified Biopolymer	Efficiency Parameter	Result	Safety Parameter	Result
[3]	In vitro/In vivo	ELP + SynB1 + doxorubicin	Tumor volume reduction	262 mm^3^ (treated) vs. 714 mm^3^ (control)	Cardiotoxicity	No fibrosis or myocardial damage
[10]	In vitro/In vivo	Cu–Mn_3_O_4_–TMC + 5-Fu	Controlled release	91.5% at 72 h, pH 5.5	Viability in healthy cells	>75%
[9]	In vitro	Eu–Fe_3_O_4_ + alginate	SAR (heating capacity)	63.5 W/g	Cell viability	51% decrease in HeLa cells
[11]	In vitro/ex vivo	HA-BSe NPs	Temperature reached	50.8 °C	Selective cell elimination	26% in tumor cells
[33]	In vitro	Chitosan + collagen + hydroxyapatite	Thermal stability	T° increased from 217 °C to 562 °C	Cell adhesion	Osteogenic differentiation confirmed
[4]	Clinical	Modulated electro-hyperthermia mEHT + chemotherapy	Tumor progression	45% (treated) vs. 24% (chemo only)	Treatment tolerance	Better tolerance in combined group
[2]	Clinical	SPIONs with coating	Induced apoptosis	90% of treated tumor volume	Systemic toxicity	No iron release detected
[34]	In vitro/In vivo	CoFe_2_O_4_ + starch and gum	SAR	72–138 W/g	Viability/hemocompatibility	97% viability/hemolysis <5%
[35]	In vitro/In vivo	FPCP (Pluronic + polypyrrole)	Muscle force	90% (treated) vs. 55% (control)	Vascularization	58 vessels/mm^2^ (vs. 33 control)
[28]	In vivo	PLGA-MXene + HA-DATS	Wound closure	99% at 7 days	Fibrosis and collagen organization	Better distribution and less fibrosis
[39]	Clinical	Alginate + MSCs	Cell viability	89.2%	Liver fibrosis	Decreased to 4.52% (vs. 33.2% control)
[40]	In vitro/In vivo	CMC + Au + tannic acid	Cell survival	90%	Inflammatory cytotoxicity	↓ IL-1β and TNF-α
[30]	In vitro	Silk fibroin/metal oxides	SAR/thermal efficiency	SAR = 72.3 W/g	Cell viability	>90% viable cells
[6]	In vitro	Chitosan/ZnO	Heating capacity	SAR 54.3 W/g	Cytotoxicity	<15% at 200 µg/mL
[32]	In vitro	PLA/metallic nanoparticles	Local thermal effect	Heating efficiency in <5 min	Cytotoxicity	>85% cell viability

**↓** indicate a decrease in the level, expression, or activity of a molecule, gene, or protein.

**Table 7:** Concentrations/doses and treatment times.

Ref.	Modified Biopolymer	Concentration/ Applied Dose	Treatment Time	Biomedical Application
[3]	Elastin-like polymer modified with doxorubicin	5 mg/kg (doxorubicin)	Every 3 days for 3 weeks	Breast cancer and cardioprotection
[2]	Magnetic iron oxide nanoparticles coated with chitosan and aminosilane	0.1–0.7 mL suspension (112 mg/mL) across 6 sessions (11.2–78.4 mg per patient)	60 min per session	Hyperthermia for glioblastoma multiforme (GBM)
[11]	Hyaluronic-acid-coated nanoparticles (HA-BSe and HDAPPs)	3.34 × 10^10^ NPs/cm^3^ in 3D models	36 s (IR irradiation); 24 h incubation	Hyperthermia and phototherapy for colorectal cancer
[28]	Photothermal hydrogel with PLGA/MXene/Nb_2_C core + HA-dopamine shell with DATS	100 ng/mg vascular endothelial growth factor (VEGF) + 50 μM diallyl Trisulfide (DATS)	7 days	Tissue regeneration, accelerated wound healing
[35]	Sodium alginate hydrogel with adipose-derived mesenchymal stem cells (MSCs)	500 μL (2% *w*/*v* alginate) with 5 × 10^6^ MSCs	7 days	Liver regeneration in induced cirrhosis

**Table 8:** Applications of thermally conductive biopolymers in regenerative medicine.

Ref.	Modified Biopolymer	Regenerated Tissue/Organ	Biomedical Application	Functional Classification
[18]	Chitosan doped with MXene and lignin	Skin (chronic wound)	Accelerated wound healing	Conductive + regenerative system
[19]	Hyaluronic acid hydrogel + PDA + CuS	Subcutaneous tissue	Wound healing	Biocompatible photothermal scaffold
[35]	PLGA + CuS and Ag nanoparticles	Soft tissue	Photothermal + antibacterial therapy	Multifunctional platform
[28]	PLGA/MXene + modified HA hydrogel + DATS	Skin (wound)	Regeneration and angiogenesis	Core–shell system with release
[36]	Thermo-responsive PNIPAM + Fe_3_O_4_ hydrogel	Liver (in vitro)	Support for cell culture	Cell-support system
[37]	Alginate scaffold + CuS nanoparticles	Skin (burns)	Thermotherapy and wound healing	Thermo-induced scaffold
[38]	Hyaluronic acid hydrogel + CNTs	Skin (infected wounds)	Photothermal and antibiotic action	Conductive hybrid system
[39]	Alginate hydrogel with MSCs	Liver	Liver regeneration	Injectable cell scaffold
[40]	Gelatin + iron oxide nanoparticles	Bone tissue	Regeneration and thermal control	Magnetically sensitive system
[41]	PLA + carbon nanofibers	Soft tissue	Photothermal wound healing	Conductive porous scaffold
[42]	Gelatin methacrylate + MoS_2_ nanosheets	Skin	Thermal, controlled healing	Regenerative photothermal hydrogel

**Table 9:** Highlighting the results of the comparative synthesis of the in vitro and in vivo applications of thermally conductive biopolymers.

Biopolymer Matrix and Composition	Modification/Fabrication Method	Biomedical Application and Treatment	Study Type/Model	Thermal Performance (Examples)	Outcome Highlights	Clinical Status
HA-DA/PLGA–MXene (Nb_2_C) composite	3D printing + photocrosslinking; core–shell designs	Vascularized bone regeneration via mild photothermal heating (~41–42 °C)	In vitro (angiogenic/osteogenic assays) + in vivo (bone model)	Mild photothermal response sufficient to trigger pro-angiogenic/osteogenic cues	Enhanced angiogenesis/osteogenesis; scaffold integration reported	Preclinical (in vitro/in vivo)
OHA + N-carboxyl chitosan + polyaniline + GSNO	Dynamic copolymerization/Schiff-base crosslinking	Diabetic wound healing with photothermal heating + NO release	In vitro (L929, HUVEC) + in vivo (murine wounds)	Photothermal response under NIR; conductive hydrogel	>90% cell viability; antibacterial; sustained NO; accelerated closure	Preclinical (in vitro/in vivo)
PCL + Au plasmonic blackbodies (AuPBs)	3D-printed composite scaffold	Bone tissue engineering with mild photothermal modulation	In vitro (bone-related cells)	Optimal 39–41 °C under NIR; mechanical reinforcement	Maintained viability; improved mechanical strength	Preclinical (in vitro)
Elastin-like polypeptide (ELP) + doxorubicin (SynB1/pHLIP)	Molecular self-assembly of drug–polymer conjugate	Breast cancer; hyperthermia-assisted targeted chemotherapy	In vitro + in vivo (4T1 murine model)	Mild hyperthermia used to enhance tumor uptake (qualitative)	Tumor volume 262 mm^3^ vs. 714 mm^3^ control; no cardiotoxicity on histology	Preclinical (in vitro/in vivo)
Alginate + metallic nanoparticles	Ionotropic gelation; in situ nanoparticle formation	Localized oncologic hyperthermia/controlled release	In vitro (+selected in vivo)	Enhanced thermal properties enabling localized heating	Controlled drug release; thermal ablation potential	Preclinical
Chitosan-coated CoFe_2_O_4_ nanoparticles	Co-precipitation + polymer coating	Magnetic hyperthermia (AMF)	In vitro (KAIMRC2 breast cancer cells) ± in vivo	Effective heating; SAR up to ~72–138 W/g (related systems)	Ferrimagnetic behavior; cytotoxicity to tumor cells under AMF	Preclinical
Alginate + flaxseed mucilage + silk fibroin + Fe_3_O_4_	Extraction + ionic gelation (CaCl_2_) + in situ Fe^2+^/Fe^3+^ co-precipitation	Hyperthermia-enabled anticancer platform	In vitro	Stable heating under AMF; good Fe_3_O_4_ dispersion	High biocompatibility; antitumor activity	Preclinical (in vitro)
Eu-doped Fe_3_O_4_ + alginate (or chitosan)	Nanoparticle embedding/coating in biopolymer	Localized magnetic hyperthermia	In vitro (HeLa cells)	SAR ≈ 63.5 W/g; efficient heating	≈51% decrease in HeLa viability at target dose	Preclinical (in vitro)
SPIONs + chitosan/aminosilane coating	Surface functionalization/coating	Magnetic hyperthermia for glioblastoma (adjunct)	Clinical: Phase I (*n* = 14), Phase II (*n* = 59)	Calibrated AMF heating in situ	Apoptosis ≈90% of treated tumor volume; no iron release reported	Clinical (Phase I–II)
Modulated electro-hyperthermia (mEHT) + chemotherapy	Regional modulated RF heating protocol	Advanced pancreatic cancer (adjunct to chemotherapy)	Clinical (multicenter, retrospective)	Regional controlled heating per protocol	Tumor control: 45% vs. 24% (chemo only); improved tolerance	Clinical (observational)
PLGA + β-TCP + MXene (Nb_2_C)	3D-printed composite; MXene doping	Vascularized bone regeneration (mild hyperthermia)	In vitro (HUVECs) + animal model	Mild heating (~41–42 °C) activates angiogenesis/osteogenesis	Accelerated vascularized bone formation	Preclinical (in vitro/in vivo)
FPCP (Pluronic F127 + poly(citric acid) + polypyrrole)	Conductive nanomatrix/hydrogel	Skeletal muscle regeneration with photothermal cues	In vitro (C2C12) + mice	On-demand thermal stimulation	Muscle force 90% vs. 55% control; vascularization 58 vs. 33 vessels/mm^2^	Preclinical (in vitro/in vivo)
PLGA–MXene core + HA-dopamine shell + DATS	Core–shell; injectable/film hydrogel	Wound healing with photothermal + NO donor (DATS)	In vivo (murine wounds)	Photothermal activation; mild hyperthermia window	Wound closure ≈99% at 7 days; better collagen/less fibrosis	Preclinical (in vivo)
Graphene + polydopamine + mupirocin (MpGel)	Conductive photothermal hydrogel	Diabetic ulcer healing; photothermal + antibacterial	In vivo (murine diabetic ulcers)	Photothermal heating under NIR	Enhanced closure; infection control; nerve regeneration signals	Preclinical (in vivo)
Alginate hydrogel + mesenchymal stem cells (MSCs)	Injectable hydrogel cell carrier	Liver regeneration in cirrhosis	Clinical (patients with cirrhosis)	—(cell therapy focus)	Cell viability 89.2%; fibrosis ↓ to 4.52% vs. 33.2% control	Clinical (pilot/feasibility)

**↓** indicate a decrease in the level, expression, or activity of a molecule, gene, or protein.

## Data Availability

Data is contained within the article.
